# Advice in Professional Economies

**Published:** 1910-10-15

**Authors:** J. P. Root

**Affiliations:** Kansas City


### ADVICE IN PROFESSIONAL ECONOMIES.

J. P. ROOT, D. D. S., KANSAS CITY.

There cannot always be new subjects of interest and profit pertaining to dentistry to write and talk about, yet those who are prolific of pen or speech must, to satisfy themselves, or their hearers, or readers, provide subjects, or be relegated to ash heap as "has beens", and far be it for them to seek such an ending. The writers of articles on any subject are not always those who supposedly should be the best informed and rightly so. It is commonly believed the writer of the Mothers' Column in the *Ladies' Home Journal* is either an old maid or a bachelor, and there is no question but what either one should be better qualified to tell how to raise children (not having any of their own, thus having more time to observe the faults of parents) than a fond and doting mother who raises her brood according to her fixed system, or lack of system, and is entirely satisfied with results, even if every one else is not, such a mother would advise all mothers to pattern after her, not realizing all children differ in disposition and temperament, and no one form of government is suitable to all.

A few years ago our magazines teemed with articles, written by prominent business men—lawyers railroad officials, etc., each telling in his simple way, how he began life without hair or teeth, and while yet in the cradle resolved to be a millionaire, or president of a trans-continental railroad, and we were told it was a simple matter of progression. James Hill, President of the Great Northern Railroad, in his article attempted to lead us to believe that ony one of the ten thousand employes under him, could by following the same tactics as he, become a railroad president, but as there is only one presidential chair connected with that corporation, opportunity would hardly allow many occupants.

The man who failed to obtain the seat would probably be better qualified to tell the other fellow how to get what he failed to.

In dental literature of today there is considerable ink used in explaining why most dentists are poor, and so few rich. The subject is of much interest to the rich ones, and to us poor ones, the reasons given are so varied it is difficult to select the real ones, which are: some of us fail to make enough, others spend too much, exactly the same reasons existing in any business or profession.

Some writers of good advice, claim we are poor because we do not charge enough. They present the deadly parallel columns, showing a carefully figured mathematical problem in percentage, giving a yearly income and expense account, in some cases basing the expense of the cost of being raised from childhood, and how much interest one should earn each year to credit to this account. They have it figured out to fractions how to eliminate all this financial worry and each and every one of us become rich, and in most cases it is by the simple process of charging more, not spending less.

I doubt if there is a class of men, of equal ability and attainments, who are better remunerated for their services than dentists, especially so when you consider the preliminary training most of them have, and the financial cost of same. The average student entering a dental college has not more than a high school education, which is not expensive. Their three years in college is not much of a luxury, except those who work their way through, and then it is hardly a luxury, and as a rule in a year or two after graduation they have an income at least double that they would have, had they been drawing a salary in some other vocation, and the increase of income as years go by is far more than that of nine out of ten salaried men. Cities are full of capable salaried men, whose early training and environments were at least equal that of ours—whose incomes are not one-half that of the average dentist, besides their working hours are longer—they are subject to rules and regulations, in fact most cases they are slaves compared to us.

I doubt if there is a vocation, where the average man has a better income than in dentistry. They are not all the same, any more than in medicine, law, or in commercial pursuits, simply because we are not all equal. He who has a clientele of millionaires naturally has an income superior to he who takes care of butchers, bakers or candlestick makers, whose incomes do not justify high prices, yet some one must do for them.

The same rule applies to all vocations, the merchant with the corner store in a residence district does not enjoy the income of he who has a department store in the business section, yet he may enjoy it more—for the size of one's income does not regulate the enjoyment of life, also we unlike the merchant are not subject to the sheriff's sale—for over ninety per cent. of all merchants fail. Go into a department store and see how many of the heads of departments are formerly prosperous merchants, compare your fate with theirs, are you compelled to be at business at eight a. m. do you push a button registering your coming? Do you repeat this on going and coming to and from lunch, and again at six p. m. when the store closes? If you are out of sorts, do you have to go to work or lose your job? If you want to go fishing on Saturday, don't you go? If you want a vacation in June or December, don't you take it, at the time you desire? Does the salaried man do these things, and would he not enjoy these privileges as much as you?

We, like the physician, policeman, preacher, merchant and everybody else, probably spend not too much, but more than we can afford, for it is a duty we owe ourselves, our family, and the world, to live within our incomes, to keep our debts paid, to provide for old age, and be satisfied with our positions in life. If dissatisfaction prevails, look around at the other fellows, not at the few who live on the hill, but at the multitude who dwell in the hollows, then thank the Lord that you are better off than most of mankind.

As I said before, the Old Maid probably can give Mothers much needed, if unsought advice, hence possibly one who is a financial failure can assist the financiers. The most consoling reflection one can have in such cases, is that he who simply chases dollars as a life occupation, is in all cases a failure in life, he may be a financial success, but wherein has he benefited mankind?—*Western Dental Journal*, for August.

